# Development of Structure–Property Relationships for Ammonium Transport through Charged Organogels

**DOI:** 10.3390/membranes14030071

**Published:** 2024-03-21

**Authors:** Adam L. Bachmann, Brock Hunter, Bryan S. Beckingham

**Affiliations:** 1 Department of Chemical Engineering, Auburn University, Auburn, AL 36849, USA; azb0274@auburn.edu; 2 Department of Chemical Engineering, The Pennsylvania State University, University Park, PA 16802, USA

**Keywords:** cation exchange membrane, sulfonic acid, nitrogen reduction

## Abstract

Ammonia is a promising carbon-free fuel, but current methods to produce ammonia are energy intensive. New methods are thereby needed, with one promising method being electrochemical nitrogen reduction cells. Efficient cell operation requires robust catalysts but also efficient membrane separators that permit the selective transport of ions while minimizing the transport of the products across the cell. Commercial membranes have an unknown morphology which makes designing improved cells challenging. To address this problem, we synthesized a series of membranes with controlled crosslinking density and chemical composition to understand their impact on ammonium transport. Higher crosslinking density led to lower ammonium permeability. At the highest crosslinking density, similar ammonium permeability was observed independent of the water volume fraction and hydrophobicity of the monomers. These results suggest new directions to develop membranes with reduced ammonium crossover to improve the efficiency of these electrochemical cells.

## 1. Introduction

Ammonia is an invaluable chemical due to its use in fertilizers and as a precursor to numerous nitrogen-containing compounds [1]. Ammonia has received renewed interest as a potential fuel source as it has one of the highest energy densities of all carbon-free fuels [2]. Notwithstanding the interest as an alternative fuel, ammonia production is a very energy-intensive process that consumes almost 2% of the world’s total energy production [3]. The vast majority of ammonia is produced using the Haber–Bosch process to react nitrogen gas with hydrogen gas under higher pressure and temperatures, generating 2.4 tons of CO_2_ per ton of ammonia [3]. To realize the development of ammonia as a carbon-free fuel, more environmentally sustainable methods of producing ammonia are needed.

Some efforts have focused on designing new catalysts that allow hydrogen and nitrogen to react at lower pressures and temperatures [4,5], while others have envisioned new reaction schemes such as chemical looping [6] or photocatalytic methods [7,8,9]. One particularly attractive method is the electrochemical nitrogen reduction reaction (NRR), which could be powered by renewable energy sources [10,11,12]. Electrochemical nitrogen reduction reacts nitrogen gas with protons and electrons generated by splitting water in the electrochemical cell, and the electricity could come from renewable sources, thereby allowing for low temperature and ambient operation. However, these methods generally suffer from a large overpotential [13], low faradaic efficiencies [14], and poor selectivity [15] for ammonia production, so many efforts are focused on designing robust electrocatalysts to improve the faradaic efficiency of the NRR [16,17,18]. One area that has received relatively little attention has been the development of tailored membranes for NRR cells. 

The cation exchange membrane (CEM), a polymer membrane that incorporates bound anions to facilitate the transport of mobile cations, separates the anodic and cathodic reactions while completing the electrical circuit by allowing cations to move between the two half cells. The ideal CEM for the NRR should demonstrate a high proton conductivity, with minimal conductivity for other ions. One of the most common membrane materials is Nafion^®^, a sulfonated fluoropolymer, which is popular because of its high conductivity and commercial availability. There is growing consensus, however, that Nafion^®^ poses two large problems to studying NRR; namely, that Nafion^®^ can be a source of ammonia contamination, while also being permeable to ammonium, the dominant form of ammonia when using acidic electrolytes [19,20,21]. Ammonium that permeates through the membrane to the anode can then be oxidized, lowering the yield of the NRR cell.

While the general structure of Nafion^®^ is known, the complex morphology of the polymer makes it difficult to develop structure–property relationships, which are needed to guide the development of new membranes [22,23]. Previous work has demonstrated the use of readily tunable (meth)acrylate gels as a platform to understand structure–property relationships in ion exchange membranes [24,25,26], with some gels demonstrating improved performance compared to commercial membranes [27]. Based on these reports, we decided to adopt this tunable chemistry to study the impact of network structure on the transport of ammonium to guide the development of new membranes for NRR cells. 

Herein, we report on the synthesis of crosslinked phenyl (meth)acrylate polymers with a sulfonic acid monomer and the transport of ammonium through these membranes. By systematically varying the amount of crosslinker in the pre-gel solution, we develop relationships between the crosslinked structure and the ammonium crossover. 

## 2. Materials and Methods

### 2.1. Materials

Phenyl methacrylate (PMA, >97%) and N,N’-methylenebisacrylamide (MBAA, >98%) were purchased from TCI (Tokyo, Japan). Phenyl acrylate (PA, 97%) was purchased from Ambeed, Inc. (Arlington Heights, IL, USA). Acrylamido-2-methyl-1-propanesulfonic acid (AMPS, 99%), ammonium chloride (NH_4_Cl), and 2,2′-azobis(2-methylpropionitrile) (AIBN, 98%) were purchased from Sigma-Aldrich Chemicals (St. Louis, MO, USA). Dimethyl sulfoxide (DMSO, ≥99%) was purchased from Macron Fine Chemicals (Radnor, PA, USA). All chemicals were used as received. High-purity nitrogen was purchased from Airgas. Type-1 deionized water (DI water) was produced by a Waterpro BT Purification System from Labconco (18.2 mΩ∙cm at 25 °C, 1.2 ppb of TOC). Nafion^®^ 117 was purchased from Chemours (Wilmington, DE, USA).

### 2.2. Membrane Formation

**Figure 1:**
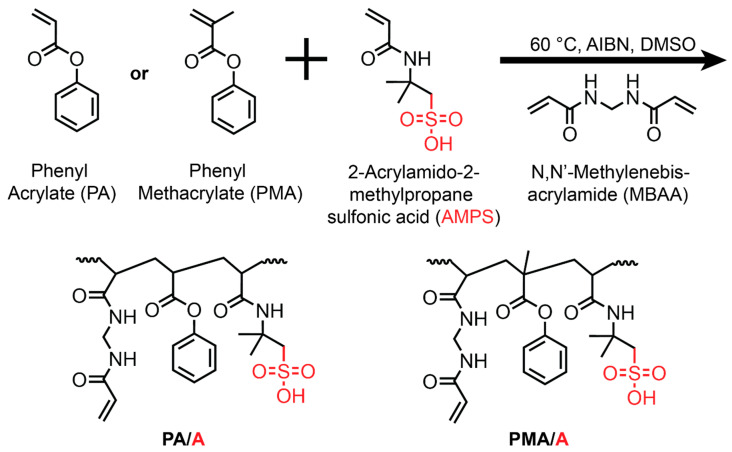
Scheme of prepared PA/A and PMA/A organogels.

Eight CEM organogels were made through the thermal copolymerization of a hydrophobic (meth)acrylate monomer, a sulfonic acid monomer, and a crosslinker (Figure 1). Four PA-AMPS and four PMA-AMPS cross-linked films were prepared. For both series, the monomer composition contained 70 mol% of the hydrophobic monomer (PA or PMA) and 30 mol% of AMPS. Each film was then crosslinked with MBAA, with the crosslinker concentration varying between 5 and 30 mol% (mol MBAA/mol total monomers). AIBN was used as the thermal initiator and was used in the amount of 0.1 wt% of the total monomer mass. Each pre-polymerization solution contained 50 wt% of DMSO. The solutions were sonicated for 15 min or until the solution was homogenous. These solutions were then purged for 10 min with nitrogen gas to remove dissolved oxygen. The mixture was spread between two glass plates (5 × 5 × 1/4″) separated by two spacers (356 μm) and put in a vacuum oven at 60 °C for 12 h. The resulting solid organogels were removed from the plates and placed in 1 L of DI water for 2 days to exchange DMSO with water; water was replaced daily (Supporting Figure S1).

### 2.3. Water Uptake, Density, and Water Volume Fraction

Water uptake was measured gravimetrically using 0.75 in. diameter punch outs from the hydrated films. The hydrated film mass, *Ws*, was measured after blotting the punch outs with tissue paper to remove adsorbed water from the surface. Films were then dried at 50 °C for 24 h under vacuum. The dried film mass, *W_d_*, was measured and water uptake, *ω_w_*, was calculated as follows:

(1) $$\omega_{w}=\frac{W_{s}-W_{d}}{W_{d}}\times 100\%$$

Film density was measured by the buoyancy method using a ML-DNY-43 Mettler Toledo density kit and a scale (ML204T, Mettler Toledo). Density, *ρ_p_*, was calculated using Equation (2), where *ρ_L_* is the density of water (997.8 kg/m^3^ at 22 °C); *ρ*_0_ is the density of air (1.225 kg/m^3^); *W*_0_ is the dried film weight in air; and *W_L_* is the weight of the film in water (Supporting Figure S2).

(2) $$\rho_{p}=\left(\rho_{L}-\rho_{0}\right)\frac{W_{0}}{W_{0}-W_{L}}+\rho_{0}$$

Water volume fraction, *φ_w_*, was then calculated as follows:

(3) $$\varphi_{w}=\frac{\left(W_{s}-W_{d}\right)/\rho_{L}}{\left(W_{s}-W_{d}\right)/\rho_{L}+W_{d}{/\rho }_{p}}$$

### 2.4. Ammonium Permeabilities

The permeabilities of each CEM and Nafion-117 to ammonium were measured at the feed concentration of 1 M. A custom-built, temperature-jacketed diffusion cell and an in situ conductivity probe (PC820 Precision Benchtop, Apera Instruments, Schaumburg, IL, USA) were used to measure the changing ammonium concentrations. The donor side cell was filled with 1 M of ammonium chloride, and the receiver cell was filled with DI water. The ammonium permeabilities, *P_i_*, were calculated using Yasuda’s model:

(4) $$P_{i}=ln\left(1-\frac{2c_{i,l}(t)}{c_{i,0}}\right)\left(\frac{-lV}{2At}\right)$$

where *c_i,l_* is the time-resolved concentration of ammonium in the receiver cell; *c_i,0_* is the initial concentration in the feed cell (1 M); *l* is the thickness of the membrane after the experiment; *V* is the volume of the half-cell (25 mL); *A* is the cross-sectional area of the exposed membrane (1.1423 cm^2^); and *t* is time. Yasuda’s model is the most established model for describing solute permeation through hydrated, swollen films, where it is assumed that the free volume is proportional to the volume fraction of water and the solute diffusivity in the water-swollen polymer is dependent on the free volume. The osmotic flow within the cell is neglected in this study, as its impact was found to be within the experimental error for an identical solution in Nafion-117.

### 2.5. Ammonium Solubility and Diffusivity

These membranes are dense polymer films, so the solution–diffusion model is used to describe the solute transport as

(5) $$P_{i}=K_{i}D_{i}$$

where *K_i_* is the solubility of the solute in the film and *D_i_* is the diffusivity of the film to the solute.

The solubility of ammonium in the films was measured using sorption–desorption experiments. From the hydrated film, 0.75 inch circles were punched out, blotted with tissue paper, and individually immersed in 15 mL of 1 M ammonium chloride solution. The punch outs were allowed to sorb ammonium chloride for 3 days with the solutions being changed daily. A digital caliper (±1 μm) was used to measure the film thickness, and the software ImageJ 1.54d (National Institutes of Health, MD, USA) was used to calculate the area of the punch outs from digital photographs after the film was saturated with ammonium chloride. The films were blotted with tissue paper after volumetric measurements and immersed in 10g of DI water for 3 days. A conductivity probe was used to measure the conductivity of the desorption solution to determine the solute concentration based on calibration experiments (Supporting Figure S3). The film solubility was calculated as follows:

(6) $$K_{i}=\frac{C_{i}^{m}}{C_{i}^{s}}$$

where *K_i_* is the solubility of the solute in the film; *C_i_^m^* is the solute concentration of the film, and *C_i_^s^* is the solute concentration of the external solution (1 M). *C_i_^m^* is determined by multiplying the solute concentration in the desorption solution by the volume of the desorption solution and dividing by the volume of the film. 

## 3. Results

A series of eight cation exchange membranes were synthesized for investigation. All eight contained the sulfonated comonomer AMPS and utilized MBAA as the crosslinker; however, four membranes contained phenyl acrylate (PA) and four contained phenyl methacrylate (PMA), enabling comparisons and an investigation of the impact of the methyl group on PMA on the physiochemical and transport properties. Within each set of four membranes, the amount of MBAA crosslinker was varied at constant monofunctional monomer ratio (30% AMPS, 70% PA or PMA); see Table 1 for membrane compositions. Once fabricated, the physiochemical properties (density, water uptake, and water volume fraction) and transport (ammonium permeability) of these membranes were characterized.

**Figure 2:**
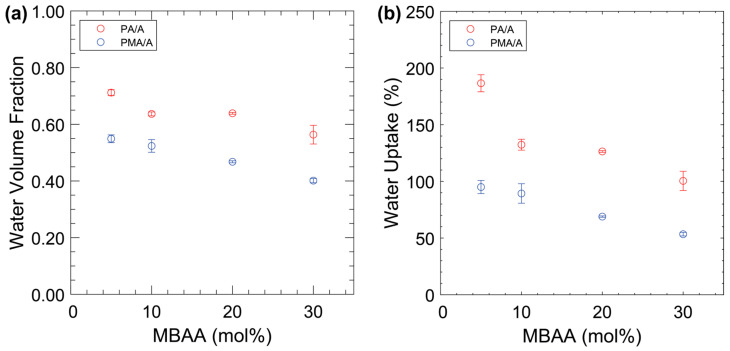
Water volume fraction (**a**) and water uptake (**b**) of PA/A (red) and PMA/A (blue) films. Each data point is the average of three membranes with error bars representing the standard deviation.

The water volume fractions and water uptakes of the eight dissimilar membranes are shown in Figure 2. The water volume fraction decreases with increasing MBAA content, which is attributed to the reduced free volume in the more densely crosslinked network. Furthermore, the PA-containing films have distinctly higher water volume fractions than the PMA-containing films. This is primarily attributed to the methyl group being both hydrophobic and much larger than the single hydrogen on PA. The measured water uptake closely mirrors the behavior of the water volume fractions, with higher water uptake for PA-containing membranes over PMA-containing membranes with decreasing water uptake with increasing MBAA content.

**Figure 3:**
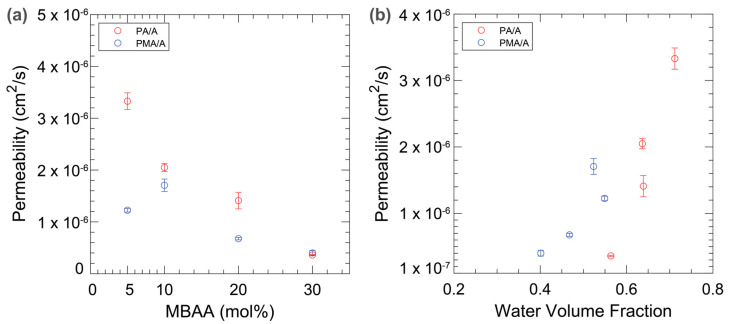
Ammonium permeabilities of PA/A (red) and PMA/A (blue) against MBAA content (**a**) and water volume fraction (**b**).

The membrane permeabilities to ammonium were measured using diffusion cell experiments and the results are shown in Figure 3 with respect to either MBAA content or water volume fraction. Increasing the MBAA content causes the permeability to decrease as it forms a denser network and slows the polymer segmental motion, which hinders diffusion through the film. Clear trends are noted for the membrane permeabilities. The dense network caused by increasing the MBAA content also limits the amount of free water in the hydrated film, which is needed to promote the facile diffusion of ammonium. Thus, the permeability drops notably as the water volume fraction decreases (Figure 3b). In this way, the increased MBAA content impacts the permeability to ammonium both by reducing the water content (where water content facilitates diffusion) and through the denser network for ammonium diffusion itself. The PMA/A films generally had lower permeabilities than the PA/A films at equivalent MBAA content, with the notable exception of the 30 mol% MBAA PA/A film. This film had a measured ammonium permeability of 3.6 ± 0.1 × 10^−7^ cm^2^s^−1^, while the PMA/A film with the same MBAA content had a permeability of 4.0 ± 0.4 × 10^−7^ cm^2^s^−1^. The similarity in permeabilities is attributed to the dense crosslinking of the system dominating the transport dynamics compared to the steric differences and/or hydrophobicity differences between PMA and PA resulting from their structures.

**Figure 4:**
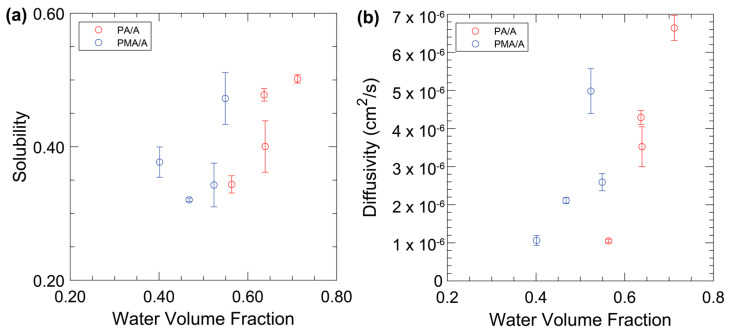
Ammonia solubilities (**a**) and diffusivities (**b**) for PA/A films (red) and PMA/A films (blue).

The solution–diffusion model (Equation (5)) says that the permeability of a species is a product of its solubility and its diffusivity. Here, we measured the solubility of ammonium in these membranes, and the diffusivity calculated based on the solution–diffusion model (Figure 4). For the solubility, there is a general positive dependence of the solubility on water volume fraction, especially for the PA/A membranes. However, we also note that the two membranes with 10 and 20% MBAA that exhibited similar water volume fractions showed dissimilar solubilities, which we attribute to the impact of MBAA itself in lieu of the differences in the crosslinked network. The film solubility to ammonia increasing with decreasing MBAA content can be explained by the higher AMPS concentration which results from this change. The sulfonated groups in AMPS increase the film solubility to charged solutes like ammonium since sulfonic acids are strong electrolytes. The PA/A films generally had higher ammonium solubilities at equivalent MBAA content, except the 30 mol% MBAA compositions.

While there is a small positive dependence of the solubility on the water volume fraction, there is a much stronger dependence for the diffusivity. While not decoupled, the stronger dependence of the diffusivity on the water volume fraction compared to the solubility suggests a potential way to engineer new membranes. A low permeability of ammonium through these membranes is achieved with dense crosslinking, as this inhibits the polymer segmental motion. The choice of hydrophobic comonomer could also be used to tune this parameter and drive the ammonium even lower than the permeability of Nafion-117, which was measured to be 1.93 ± 0.04 × 10^−7^ cm^2^s^−1^.

## 4. Conclusions

A series of CEMs were prepared and tested for applications in NRR cells. As the cross-linker content increased, the ammonium permeability, solubility, and diffusivity generally decreased. Two hydrophobic comonomers were tested, PMA and PA, and PMA generally showed lower ammonium permeability than PA, although the PA film with 30 mol% MBAA content achieved the lowest permeability of the films tested. This work quantified the weak dependence of solubility on crosslink density and the strong dependence of diffusivity on crosslink density. This work will provide guidance on the development of future membranes for NRR cell applications to reduce ammonium crossover. 

## Figures and Tables

**Table 1:** Membrane compositions.

Name	PA (mol%)	PMA (mol%)	AMPS (mol%)	MBAA (mol MBAA/mol Total Monomers)
PA-5	70	0	30	5
PA-10	70	0	30	10
PA-20	70	0	30	20
PA-30	70	0	30	30
PMA-5	0	70	30	5
PMA-10	0	70	30	10
PMA-20	0	70	30	20
PMA-30	0	70	30	30

## Data Availability

The data presented in this study are available on request from the corresponding author. The data are not publicly available due to ongoing research using a part of the data.

## Supplementary Materials

The following supporting information can be downloaded at: https://www.mdpi.com/article/10.3390/membranes14030071/s1, Figure S1: Photos of film as an organogel (left) and punch out (right); Figure S2: Film Densities for PA (red) and PMA (blue); Figure S3: Calibration plot of measured conductivity versus concentration for aqueous ammonium chloride; Table S1: Water uptakes, film densities, and water volume fractions for PA and PMA films. Number in film name indicated MBAA content; Table S2: Ammonium permeability, solubility, and diffusivity for PA and PMA films.
