# Supplementary material for: Development of Structure–Property Relationships for Ammonium Transport through Charged Organogels
Source: Membranes (Basel). 2024 Mar 21;14(3):71. doi: 10.3390/membranes14030071 (PMC10971853; doi:10.3390/membranes14030071)
Supplement: Supplementary file 1 [file membranes-14-00071-s001.zip › membranes-2823561-supplementary.pdf]

Supporting Information

Development of Structure-Property Relationships for Ammonium Transport Through Charged Organogels

Adam L. Bachmann, Brock Hunter, Bryan S. Beckingham

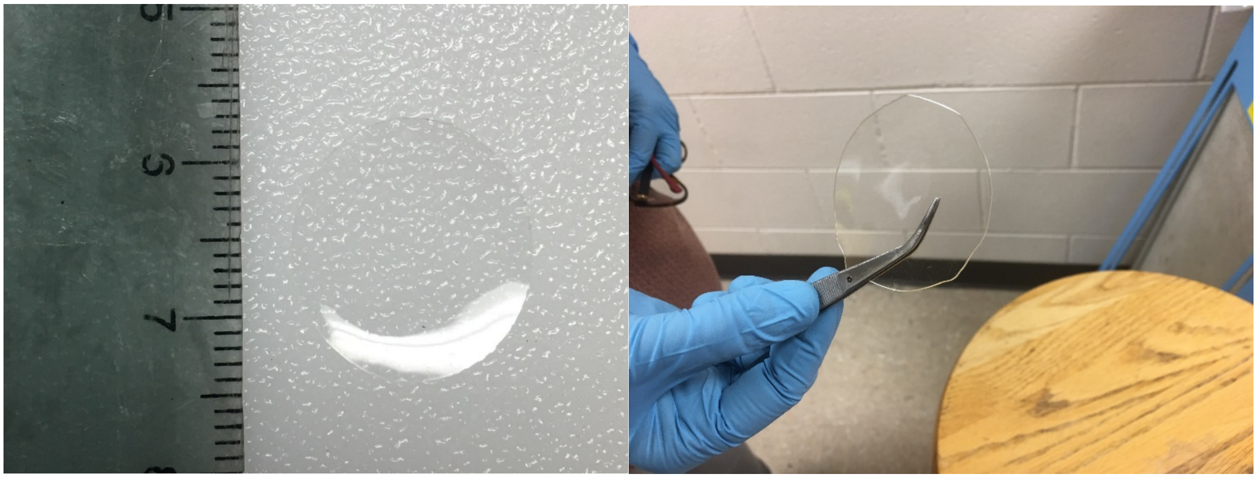

Figure S1. Photos of film as an organogel (left) and punch out (right).

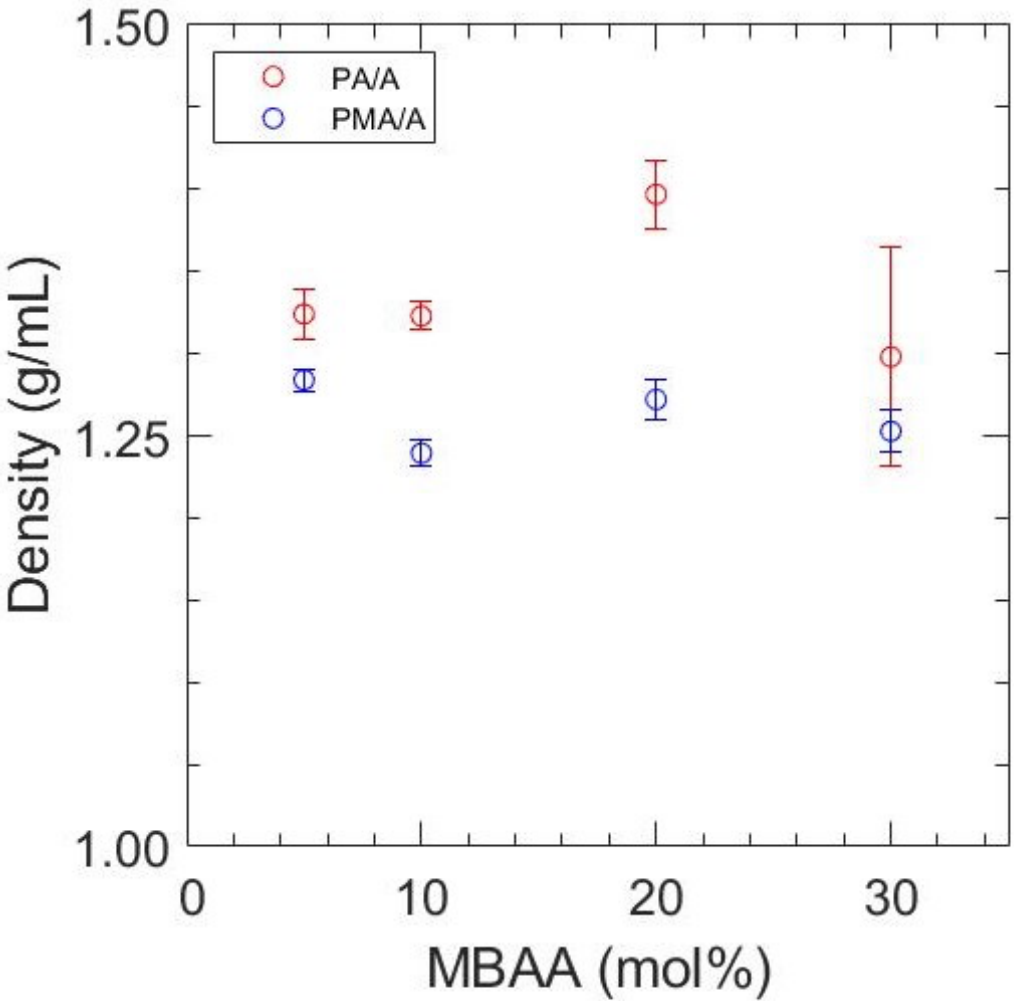

Figure S2. Film Densities for PA (red) and PMA (blue).

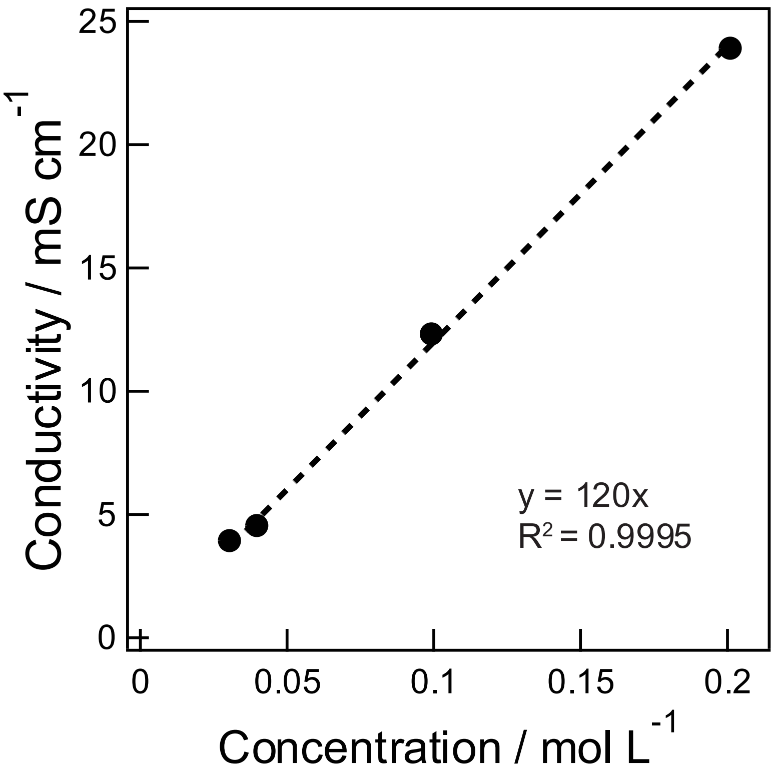

Figure S3. Calibration plot of measured conductivity versus concentration for aqueous ammonium chloride

Table S1. Water uptakes, film densities, and water volume fractions for PA and PMA films. Number in film name indicated MBAA content

Name	Water Uptake (%)	Film Density (g/mL)	Water Volume Fraction
PA-5	186 ± 8	1.32 ± 0.02	0.71 ± 0.01
PA-10	132 ± 5	1.323 ± 0.009	0.637 ± 0.007
PA-20	126.4 ± 0.8	1.40 ± 0.02	0.645 ± 0.003
PA-30	100 ± 8	1.30 ± 0.07	0.56 ± 0.03
PMA-5	95 ± 6	1.284 ± 0.007	0.55 ± 0.01
PMA-10	89 ± 9	1.24 ± 0.01	0.52 ± 0.02
PMA-20	68.9 ± 0.6	1.272 ± 0.008	0.468 ± 0.004
PMA-30	53 ± 2	1.25 ± 0.01	0.401 ± 0.008

Table S2. Ammonium permeability, solubility, and diffusivity for PA and PMA films.

Name	Permeability ($\times10^{-7}$ cm s^{-1})	Solubility	Diffusivity ($\times10^{-7}$ cm s^{-1})
PA-5	33 ± 2	0.502 ± 0.006	66 ± 3
PA-10	20.5 ± 0.8	0.477 ± 0.009	43 ± 2

PA-20	14 ± 2	0.40 ± 0.04	35 ± 5
PA-30	3.6 ± 0.1	0.34 ± 0.01	10.5 ± 0.5
PMA-5	12.3 ± 0.4	0.47 ± 0.04	26 ± 2
PMA-10	17 ± 1	0.34 ± 0.03	50 ± 6
PMA-20	6.8 ± 0.2	0.320 ± 0.002	21.1 ± 0.7
PMA-30	4.0 ± 0.4	0.38 ± 0.02	11 ± 1
